# Immune Response to COVID-19: Can We Benefit from the SARS-CoV and MERS-CoV Pandemic Experience?

**DOI:** 10.3390/pathogens9090739

**Published:** 2020-09-09

**Authors:** Emilia Sinderewicz, Wioleta Czelejewska, Katarzyna Jezierska-Wozniak, Joanna Staszkiewicz-Chodor, Wojciech Maksymowicz

**Affiliations:** 1 Department of Neurosurgery, Laboratory of Regenerative Medicine, School of Medicine, Collegium Medicum, University of Warmia and Mazury in Olsztyn, 10-082 Warszawska, Poland; wioleta.czelejewska@uwm.edu.pl (W.C.); katarzyna.jezierska@uwm.edu.pl (K.J.-W.); joanna.chodor@uwm.edu.pl (J.S.-C.); 2 Department of Neurosurgery, School of Medicine, Collegium Medicum, University of Warmia and Mazury in Olsztyn, 10-082 Warszawska, Poland; wojciech.maksymowicz@uwm.edu.pl

**Keywords:** COVID-19, immune system, MERS, SARS, SARS-CoV-2

## Abstract

The global range and high fatality rate of the newest human coronavirus (HCoV) pandemic has made SARS-CoV-2 the focus of the scientific world. Next-generation sequencing of the viral genome and a phylogenetic analysis have shown the high homology of SARS-CoV-2 to other HCoVs that have led to local epidemics in the past. The experience acquired in SARS and MERS epidemics may prove useful in understanding the SARS-CoV-2 pathomechanism and lead to effective treatment and potential vaccine development. This study summarizes the immune response to SARS-CoV, MERS-CoV, and SARS-CoV-2 and focuses on T cell response, humoral immunity, and complement system activation in different stages of HCoVs infections. The study also presents the quantity and frequency of T cell responses, particularly CD4^+^ and CD8^+^; the profile of cytokine production and secretion; and its relation to T cell type, disease severity, and utility in prognostics of the course of SARS, MERS, and COVID-19 outbreaks. The role of interferons in the therapy of these infections is also discussed. Moreover, the kinetics of specific antibody production, the correlation between humoral and cellular immune response and the immunogenicity of the structural HCoVs proteins and their utility in the development of a vaccine against SARS, MERS, and COVID-19 has been updated.

## 1. Introduction

Coronaviruses (CoVs) were discovered in the 1930s as zoonotic spherical pathogens causing mostly respiratory or enteric diseases [1,2]. Coronaviruses vary in size and are enveloped with club-shaped spikes on their surface [3,4,5]. A helically symmetrical nucleocapsid comprising positive-sense single-stranded RNA is one of the largest virus genomes, ranging from 26 to 32 kilobases in length [6]. Although CoVs are distributed mainly among mammals and birds, over the last 20 years some CoV infections have resulted in lethal epidemics in humans.

Since 1960, when the first human coronavirus (HCoV) was identified, seven HCoVs species have been described [7]. Four of them, HCoV-229E, HCoV-OC43, HCoV-NL63 and HCoV-HKU1, lead to mild diseases such as the common cold, while the SARS-CoV, MERS-CoV, and SARS-CoV-2 caused severe disorders, manifesting acute respiratory system failures and fatalities [8]. The first identified HCoV, SARS-CoV, originated from southern China in 2003 and induced an epidemic of Severe Acute Respiratory Syndrome (SARS) with a mortality rate of 10–15% [9,10,11]. The first case of MERS-CoV, inducing Middle East Respiratory Syndrome (MERS), was reported in Saudi Arabia in 2012. The fatality rate of MERS was estimated at 34.4% [12,13,14]. The most recent HCoV causing severe pneumonia, first detected in Wuhan City, Hubei Province, China, was reported to the World Health Organization (WHO) in December 2019 [15]. Next-generation sequencing of the viral genome showed high homology to the SARS-CoV and MERS-CoV (79% and 50%, respectively) [11,16,17,18]. According to its phylogenetic tree and taxonomy analysis, the virus was identified as severe acute respiratory syndrome coronavirus 2 (SARS-CoV-2) and SARS-CoV-2-associated severe respiratory disease was called Coronavirus Disease-19 (COVID-19) [19]. Although pathogenic HCoVs, including the bat-derived CoV-like coronaviruses (the source of SARS-CoV-2) originated from different animal hosts, all of them are classified as being part of the β-CoV genera [7,11].

Although there are similarities between the genome sequences of SARS-CoV, MERS-CoV, and SARS-CoV-2, the transmission force and spectrum of diseases caused by the above HCoVs seem to be different. The fatality rate of COVID-19 in June 2020 oscillated around 5.3%; however, the changing scale of the pandemic may influence this ratio. Transmission of SARS-CoV-2 is more effective than in SARS-CoV or MERS-CoV because of human-to-human SARS-CoV-2 transfer [20,21], but the transmission ways are the same [22,23]. Moreover, virus transfer occurs independently of the onset of symptoms [24,25]. The presence of an intermediate host of SARS-CoV-2 facilitating the emergence of the virus in humans also cannot be excluded, such as civet cats being intermediate hosts for SARS-CoV and dromedary camels for MERS-CoV [26,27,28,29].

The presence of the pathogens generates an immune response in the host organism, directed against the structural components of the extraneous unit. The structure of HCoVs, including SARS-CoV-2, has been widely reviewed in the literature [7,30]. Among the principal structural proteins, common for all HCoVs, the most involved in effective infection and related to immune response are envelope (E) and the nucleocapsid (N) proteins, which participate in viral assembly and budding, and the spike (S) protein, binding to the specific receptors present in the host cells [31,32,33]. It has also been documented that the structure of SARS-CoV-2 receptor-binding domain is similar to that of SARS-CoV [34,35,36,37]. Although both cell-mediated and humoral immune responses generated against the structural proteins of SARS-CoV and MERS-CoV have been reported, the immunological information about SARS-CoV-2 remains poorly described and incomplete.

The similarities between SARS-CoV and SARS-CoV-2, manifested in high genome homology, mechanism of cell admission, and connection to specific human receptors, allow an easier understanding the pathomechanism of SARS-CoV-2 action and its influence on the immunological system of COVID-19 patients. Furthermore, the experience acquired within previous epidemics of SARS and MERS, having similar clinical symptoms and course of the disease to COVID-19, may provide a useful tool in determining the treatment and support vaccine development. This paper provides an analysis of the immunogenicity of SARS-CoV, MERS-CoV, and SARS-CoV-2 infections and a review of the types of immune responses to SARS, MERS, and COVID-19.

## 2. Cell-Mediated Immunity

### 2.1. T Cell Response to HCoVs

The first observed effects of HCoV infections concerned a strong specific T cell response. In SARS-CoV infection, a rapid reduction of T lymphocytes, both CD4^+^ and CD8^+^ in peripheral blood was observed, often even before any abnormal changes were observed in chest X-rays [38,39]. A multivariate regression analysis showed that SARS severity strongly correlated with a high level of CD4^+^ T cell response, but not with CD8^+^. On the other hand, the analysis of T cell subpopulations in SARS patients showed greater quantity and frequency of the CD8^+^ T cell responses in comparison with CD4^+^ [40]. The CD8^+^ T cell action was presented across the whole SARS proteome, while the CD4^+^ T cell responses were mainly typical for the S protein. Moreover, the SARS-CoV-specific CD4^+^ T cells from the severe group of patients aimed to be a central memory phenotype (CD27^+^/CD45RO^+^) [40]. It was also found that the total number of T lymphocytes, CD3^+^, CD4^+^, and naive CD4^+^ T cells was still lower one year post-SARS-CoV infection compared to the control value [41]. Only CD8^+^ T cells returned to normal level in the recovery period, probably as the effect of recirculation between blood and organs [41]. The ability of the MERS-CoV to infect T cells and the activation of extrinsic and intrinsic apoptosis pathways in T cells was also proven [42]. Similar to SARS-CoV infection, effective transmission of MERS-CoV resulted in downregulation of Th2 and high frequencies of reactive CD8^+^ T cells in the first phase of the disease, but not in the convalescent phase [43,44]. Furthermore, a correlation between Th1 and Th2 downregulation and the fatality rate of MERS-CoV and SARS-CoV infection was found [40,43,44,45]. The newest reports have also documented an assessment of the T cell numbers in COVID-19 patients. Similar to SARS and MERS, the number of total T cells, CD4^+^, and CD8^+^ T cells was significantly diminished in COVID-19 patients in comparison to healthy controls and positively correlated with the severity of the disease [46,47]. Moreover, an age-dependent reduction in T cell numbers was observed in COVID-19, with the lowest T cells numbers detected in individuals older than 60 years old, indicating an enhanced susceptibility to SARS-CoV-2 infection in elderly patients. Furthermore, besides the decreasing number of T cells, the limited function of these cells has been described as a result of enhanced expression of immune-inhibitory factors, such as programmed death receptor 1 (PD1) or hepatitis A virus cellular receptor 2 (Tim-3) [47]. A flow cytometry analysis illustrated significantly greater expression of the PD1 and Tim-3 on T cell surfaces isolated from COVID-19 patients in comparison to healthy controls [47]. Growing PD1 and Tim-3 levels were found as patients progressed from prodromal to overtly symptomatic stages, suggesting that the surviving T cells lost their functionality, particularly in patients requiring Intensive Care Unit care. Moreover, stimulation of peripheral blood mononuclear cells (PBMCs) from group of severe COVID-19 patients with peptides covering all viral proteins activated both CD4^+^ and CD8^+^ SARS-CoV-2-specific T cells [48]. Furthermore, a greater CD4^+^:CD8^+^ ratio in COVID-19 patients was observed in comparison with control individuals [48]. Similar to SARS-CoV, SARS-CoV-2-specific CD4^+^ T cells were identified as central memory T cells, based on CD45RA and CCR7 expression. A mixed phenotype of CD8^+^ T cells in COVID-19 patients was also documented [48]. A low content of CD3^+^ T cells in peripheral blood of COVID-19 patients and a negative relationship between viral and CD4^+^ titers were also reported [48]. Moreover, SARS-CoV-2-specific CD8^+^ and CD4^+^ T cells were detected in ~70% and 100% of COVID-19 convalescents, respectively [49]. The response of CD4^+^ T cell to SARS-CoV-2 S protein was also correlated with the specific IgG and IgA titers in patients who recovered from COVID-19 [49].

Li et al. [40] established detailed maps of T cell immune responses to SARS-CoV using PBMCs from SARS convalescents. The 55 new T cell epitopes were identified which induced a response to eight of fourteen SARS proteins: replicase, Orf3, Orf4, Orf13, spike (S), envelope (E), membrane (M), and nucleocapsid (N). Almost 70% of the responses were focused on structural proteins (S, E, M, and N), principally on S protein (41%), whereas the most abundant in SARS-CoV proteome replicase was much less immunogenic [31,40].

Immune-informatic tools were used to identify significant cytotoxic T lymphocyte (CTL) and B cell epitopes in the SARS-CoV-2 surface glycoprotein [50]. Ahmed et al. [51] documented S and N SARS-CoV-2 protein-derived epitopes, which were comparable to the SARS-CoV map B cell and T cell epitopes. The surface glycoprotein of SARS-CoV-2 was found to have 76.3% identity and 87.3% similarity to SARS-CoV [50]. Moreover, five CTL epitopes, three sequential B cell epitopes, and five discontinuous B cell epitopes in the viral surface glycoprotein were detected and described [50]. Despite their high similarity to SARS-CoV, 12 of 13 identified sequential CTL and B cell epitopes were at least partially unique to SARS-CoV-2 compared with Bt-CoV, SARS-CoV, and MERS-CoV. A molecular dynamics stimulation showed that all CTL epitopes bind strongly to the peptide-binding groove of corresponding MHC class I molecules [50]. These features of CTL epitopes suggested their potential in induction of immune responses and, thereby, utility in a vaccine against SARS-CoV-2.

The T cell responses against the S and N proteins were documented as the most long-term reaction in SARS-CoV-infection [52]. Similar results, showing strong specific T cell response against structural proteins, including S, N, M, and E proteins, were noted in MERS-CoV infections [53,54,55,56]. Specific SARS-CoV-2 S and N proteins were also documented as the most immunogenic and greatly expressed during COVID-19 [48,50,51]. Although CD4^+^ T cell activation was reported against S, M, and N proteins, as well as against nsp3, nsp4, ORF3a, and ORF8, only S protein induced a robust response [49]. Activation of CD8^+^ T cells was detected against SARS-CoV-2 S and M proteins and at least eight ORFs [49]. Similar results were presented by Le Bert et al. [57], who proved the reactivity of both CD4^+^ and CD8^+^ T cells to the N protein and non-structural (NSP7 and NSP13 of ORF1) proteins of SARS-CoV-2 in COVID-19 convalescents. It was also shown that SARS convalescents responded to the N protein of SARS-CoV-2 [57]. Uninfected individuals also revealed SARS-CoV-2-specific CD4^+^, indicating possibility of cross-reactive T cell stimulation with the other HCoVs [49,57]. Interestingly, SARS-CoV-2-specific T cells in controls expressed a different pattern of immunodominance in comparison to SARS and COVID-19 convalescents [57]. Patients who recovered from SARS or COVID-19 responded mainly to N protein, whereas the control group revealed dominant reactivity to both N protein and ORF1-encoded proteins [57].

### 2.2. Cytokines Secretion in HCoV Infections

Cytokines, produced mainly by immune cells like macrophages, B and T lymphocytes, and mast cells, modulate the balance between humoral and cell-based immune responses [58]. Their concentration in biological fluids may be an important marker of immune system activity and disease progress. Cytokines include several protein groups which vary in function, cell secretion, and target action. The current study reviewed the role of interleukins (ILs) with tumor necrosis factors (TNFs), chemokines and interferons (IFNs) in the immune response to HCoVs.

#### 2.2.1. Interleukins and Tumor Necrosis Factors

A comparison of the content of proinflammatory Th1 and Th2 cytokines in the serum of SARS patients with healthy controls documented a significantly greater concentration of TNF-α, IL-6, IL-8, IL-10, and IL-12 in the early stage of the SARS-CoV infection [32,40]. Decreasing levels of these cytokines were correlated with the course of recovery from SARS-induced pneumonia. Furthermore, significantly greater contents of IL-4, IL-5, and IL-10 were reported in fatal SARS cases [40]. The enhanced secretion of IL-1α, IL-1β, IL-6, IL-8 IL-12, and IFN-γ as an antiviral and inflammatory response to MERS-CoV was also documented [43,44,59,60]. Moreover, IL-8 and IL-12 were produced in a greater amount in response to MERS-CoV than SARS-CoV [59]. Interestingly, in vitro studies showed that enhanced IL-6 and IL-8 levels in SARS and MERS patients were observed exclusively in the presence of S protein [32,60].

Among the cytokines involved in the immune response against HCoVs, several have been proposed as potential predictors of disease cause and progression. It has been documented that increased IL-6 concentration in plasma of SARS patients was significantly increased in severe cases, but not in convalescent or control subjects, suggesting a positive correlation between serum IL-6 level and disease severity [61]. Inversely, IL-8 and TGF-β concentrations were significantly reduced in SARS patients with a severe course of the disease [61]. TNF-α was considered a predictor of disease progression due to its greatest level in the early stage of recovery [32]. Moreover, a decreased content of IL-4 and increased level of IL-10 were only found in convalescent patients [61]. It was also proven that the IL-8 profile in patient’s serum indicated the cause of pneumonia—a significantly lower IL-8 concentration was detected in SARS patients compared to others.

A detailed analysis showed that in a group of SARS patients with severe symptoms, cytokine secretion was varied among different T cell subpopulations. It was shown that IFN-γ and TNF-α were produced both by CD8^+^ and CD4^+^ T cells, whereas the production of IL-2 was typical exclusively for CD4 ^+^ [40]. Moreover, in this group of patients the number of polyfunctional memory CD4^+^ T cells producing more than one cytokine was significantly higher compared to SARS patients with a mild or moderate course of disease [40]. A similar effect was not observed for CD8^+^ T cell responses, although intensified degranulation was observed in severe course of SARS via CD107a activation on CD8^+^ T cells surface [40]. Stimulation of PBMCs from recovered SARS patients with peptides overlapping the entire E protein, a membrane component of SARS-CoV, resulted in cytokine production by both CD4^+^ and CD8^+^ T cells [31].

Similar observations have been reported in studies concerning COVID-19. The lack of expression of the receptor for SARS-CoV-2 on T cells, ACE2, suggested that the limited T cell number in COVID-19 patients was likely caused by the influence of cytokine signaling and not by the direct infection of T cells [47,62]. The stimulation of PBMCs from COVID-19 patients with S protein peptides resulted in production of effector or Th1 cytokines (IFN-γ, TNF-α, and IL-2) and, to a lesser extent, Th2 (IL-5, IL-13, IL-9, and IL-10) and Th17 (IL-17A, IL-17F, and IL-22) cytokines [48]. However, among the numerous serum cytokines, only TNF-α, IL-1, IL-6, and IL-10 levels were significantly increased in SARS-CoV-2 infected patients [46,47,63]. These changes were characteristic of severe progression of the disease, supporting the hypothesis that COVID-19 is driven by proinflammatory cytokines, which are responsible for histological changes and clinically full-blown cases of the disease. Among detected cytokines, IL-6 appears to be the most significantly involved in COVID-19 progress. Chen et al. [46] detected an enhanced level of IL-2R in severe cases of COVID-19, although no significant differences among examined and control groups were detected in IL-2 [47]. The presence of IL-4, greatly expressed in fatal SARS cases, was also not detected in the plasma of COVID-19 patients [47]. Moreover, the concentration of TNF-α, IL-6, and IL-10 was negatively correlated with amounts of total T cells, CD4^+^ T cells, and CD8^+^ T cells, respectively. Furthermore, serum concentrations of IL-10, IL-6, and TNF-α were significantly lower in patients in the disease resolution in comparison to the disease period, whereas the total number of T cells, CD4^+^ T cells, and CD8^+^ T cells was restored during the decline period of COVID-19. These results suggested that in SARS-CoV-2 infections, a high serum concentration of TNF-α, IL-6, and IL-10 negatively regulated T cell survival and/or proliferation [47]. Interestingly, the production of cytokines by CD4^+^ (mainly IL-2 and IFN-γ and trace amounts of IL-4, IL-5, IL-13, or IL-17α) was also reported in COVID-19 convalescents [49]. Thus, the functional response of CD4^+^ against SARS-CoV-2 was suggested in recovered patients.

#### 2.2.2. Chemokines

Chemokines are essential in determining immune cell localization [64], and some of them act as factors involved in response to HCoVs. Enhanced contents of IP-10/CXCL-10, MCP-1/CCL-2, MIP-1α/CCL-3, and RANTES/CCL-5 were identified in the lungs and peripheral blood of SARS and MERS patients [45,59,65,66]. Moreover, both the production and secretion of these molecules were greater in response to MERS-CoV in comparison to SARS-CoV [59]. The upregulation of CXCL-10 at both transcriptional and translational levels was proven in murine epithelial cells, lung fibroblast cells, monocyte-derived macrophages, and dendritic cells as a result of the overexpression of MERS-CoV N protein [33]. High secretion and a persistent increase of CXCL-10 in MERS-CoV patients were associated with disease severity [60]. MERS-CoV infection also resulted in CXCL-8 chemokine production by Th1 cells [43,44]. The presence of chemokines and their action has not been reported in COVID-19, although the number of studies concerning SARS-CoV-2 infection is still limited.

#### 2.2.3. Interferons

Among crucial elements of the immediate antiviral response, interferons (IFNs) are pivotal for limiting viral replication and spread. Therefore, IFNs were extensively studied as potential therapeutic tools of SARS-CoV and MERS-CoV infections. The presence of an enhanced level of IFN-γ was documented in sera of SARS-CoV- and MERS-CoV-infected patients [40,59]. Similar to the other cytokines, the IFN-γ profile was correlated with the cause of pneumonia. IFN-γ production was significantly greater in SARS patients compared to others [61]. On the other hand, further studies documented relatively low IFN-γ production in response to SARS-CoV infection. Zhou et al. [59] found greater levels of IFN-γ in sera of MERS patients compared to SARS-CoV-infected patients. Moreover, Scagnolari et al. [67] showed that IFN-γ production in response to SARS-CoV was significantly lower compared to well-established IFN-inducing viruses, such as vesicular stomatitis (VSV) and Newcastle viruses (NDV), suggesting a limited role of IFNs in early host defense against SARS-CoV infection. The lack of the antiviral IFN response to SARS-CoV with simultaneous enhanced secretion of several proinflammatory cyto- and chemokines suggested that the virus suppresses the induction of IFN production [65]. The natural host defense based on IFN action may be restricted because of the documented inhibition of IFNs type I and cytokines production in toll-like receptor (TLR) 3, TLR7, and retinoic acid-inducible gene 1 (RIF-I) pathways in response to SARS-CoV infection. This limitation occurs via suppressing the activation of transcription factors, such as interferon regulatory factor 3 (IRF3), nuclear factor (NF)-κB, and adaptor related protein complex 1 (AP1) and downregulation of TNF receptor associated factor (TRAF) 3 and TRAF6 [68]. It was also documented that MERS-CoV N and M proteins inhibited the gene expression of IFNs type I and III, resulting in the host antiviral response impairment [55,56].

Similar results were documented for SARS-CoV-2 infection. Chen et al. [46] showed the production of IFN-γ by CD4^+^ T cells in response to SARS-CoV-2 tended to be lower in severe (14.1%) than in moderate (22.8%) cases of COVID-19. However, among the very few reports concerning the role of IFNs in COVID-19 disease was a study documenting a lack of IFN-γ in the serum of patients infected with SARS-CoV-2 [47].

On the other hand, further studies documented the utility of IFNs in the treatment of SARS-CoV infection. Chen et al. [65] confirmed virus susceptibility to exogenous type I IFN. It was also shown that early administration of IFNs-I decreased immunopathological changes via downregulation of the expression of factors inducing apoptosis; upregulation of hypoxia/hyperoxia-related genes and the regulation of TLR, cytokine, and chemokine signaling; and expression of MHC-, lysosome-, and fibrosis-related genes [69,70]. However, high-level virus replication resulted in retardation of IFNs-I signaling, which promoted the cumulation of pathogenic inflammatory monocyte macrophages and resulted in increased cytokine and chemokine levels in lungs, vascular leakage, and reduced virus-specific T cell responses, and thereby strong lung pathology. Animal models showed that genetic ablation of IFN-α/β receptor (IFNAR) depletion protected from lethal infection, without affecting viral load [70], suggesting that IFNs therapy may be effective mainly in the early stage of infection.

It was also shown that IFNs-I and TLR agonists were the most effective in SARS and MERS therapy, which activates interferons [66]. The best results were observed for IFN-β1a, which reduced mortality by 20% in comparison to patients, who received IFN-α2a. The efficacy of IFNs was lower in older patients [68]. The ability to induce IFNs mRNA accumulation by SARS-CoV in PBMCs from healthy donors was also investigated by Castilletti et al. [71], who proved that combination of IFN-α and IFN-γ strongly inhibited virus replication, while single cytokines were much less effective.

#### 2.2.4. Cytokine Receptors and Ligands

**Figure 1:**
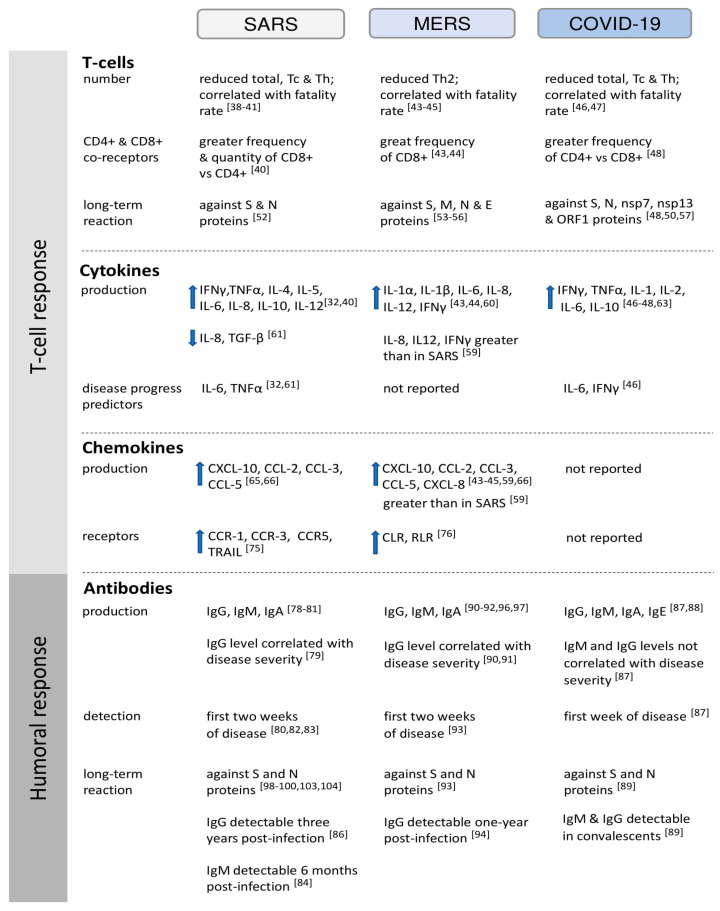
Characteristics of immune responses in SARS, MERS, and COVID19 disease. Figure 1 presents the main aspects of T cell and humoral responses in human coronaviruses infections. Arrows indicate increased or reduced expression of chosen factors in SARS, MERS, and COVID-19 patients in comparison with controls. Parentheses comprise references for presented data.

An analysis of molecular mechanisms of the immune response to HCoVs showed that effective cytokine production correlated to the availability of functional HCoVs receptors. The effective increase in IL-8 level was similar to concentration observed for S protein binding to SARS-CoV functional receptor, ACE2, or to neutralizing monoclonal antibody. It was documented that IL-8 production also depended on NF-κB activation and translocation and was suppressed by an NF-κB inhibitor [32]. Moreover, the latest studies suggested that protein S could activate PBMCs via the TLR2 ligand. It was demonstrated that a lack of functional TLR3, TLR4, and TLR adaptor molecule 2 (TRAM) enhanced the possibility of SARS-CoV infection, reduced lung function and increased lung pathology and mortality. The suppression of TLR adaptor molecule 1 (TRIF) in mice infected with SARS-CoV resulted in changes in inflammation and positively correlated with acute respiratory distress syndrome [72]. On the other hand, infection of macrophages with MERS-CoV resulted in a reduced capacity to produce TNF-α and IL-6 and enhanced the IL-10 secretion [73]. The role of MERS-CoV S protein in upregulation of the IRAK-M expression, which is a negative regulator of TLR signaling, as well as expression of the transcriptional repressor PPAR-γ was documented. Moreover, it was documented that the immunosuppressive effect was mediated by dipeptidyl peptidase 4 (DPP4), which competitively inhibits MERS-CoV via binding to common for MERS-CoV and DPP4 functional receptor, DPP4R [73,74]. In human dendritic cells (DC), the induction of C-C motif chemokine receptor (CCR) 1, CCR3, and CCR5 in the presence of SARS-CoV was detected [75]. The SARS-CoV infection induced also significant upregulation of TNF-related apoptosis-inducing ligand (TRAIL) gene expression in DCs [75]. It was demonstrated that, in MERS-CoV infection, C-type leptin receptor (CLR) was also upregulated and a retinoic acid-inducible-I-like receptor (RLR) pathway was activated [76]. The main aspects of T cell response in HCoVs infections are shown in Figure 1.

## 3. Humoral Immunity

### 3.1. Kinetics of Antibody Production in Response to HCoVs

Humoral immune response restrains the infection via neutralizing antibodies production and prevents reinfection in the future [77]. In SARS, the presence of IgG, IgM, and IgA antibody responses was detected during both the infection and convalescent phases, although with variable dynamics [78,79,80,81]. The presence of specific IgG and IgM antibodies was also documented in the first two weeks of the SARS-CoV infection (59.1% and 36%, respectively) [78,80,82,83]. The levels of IgG and IgM increased during the next two weeks to 97% and 82%, respectively. The serum samples examined 25 days after the onset of disease were positive only for SARS-specific IgG [78,83]. A study analyzing the kinetics of specific antibody contents in plasma of SARS patients presented by Mo et al. [84] also showed a further significant increase in IgG antibody levels. The highest concentration of IgG was documented on day 60, remaining at the same level until day 180. Then, the IgG content gradually decreases until day 540. The IgM antibody level peaked shortly after its detection and, in contrast to previous studies, declined until day 180 when IgM was undetectable [84]. Similar results were found by Chen et al. [79], who suggested that SARS-CoV-specific IgG antibody, persisting for a longer time than specific IgM and IgA antibodies, was the primary humoral immune response against SARS. However, a significantly lower level of IgG was detected in severe than in mild or recovering SARS patients, which may be a result of some kind of immune system dysfunction in long-suffering acute patients. However, Li et al. [40] reported that strong T cell responses correlated significantly with a higher level of neutralizing antibody activity. In contrast to memory T cell responses, ensuring long-term protection, the antibody response was transient in convalescent SARS patients [85]. Cao et al. [86] documented the presence of specific antibodies within three years from the onset of SARS symptoms in 94.7% of examined samples. However, six years post-infection, SARS-CoV-specific IgG and Ag-specific memory B cells were undetectable in SARS convalescents, whereas memory T cell responses to a pool of SARS-CoV S peptides were revealed in 60% of convalescents. The most recent study reported presence of long-lasting memory T cells responding to SARS-CoV N protein in SARS convalescents 17 years after the SARS pandemic [57]. Moreover, memory T cell response was stronger in former patients, who revealed severe clinical manifestations during SARS [85]. Similar to SARS-CoV infection, IgM and IgG levels increased during the first week after SARS-CoV-2 infection. The greatest concentration of IgM was detected in the second week, after which its content was reduced to initial level in most patients, whereas the IgG level remained at a high level for a long period [87]. Interestingly, the IgM and IgG antibody levels were not significantly different among mild, severe, and critical disease groups [87]. However, the levels of IgG, IgA, and IgE were greater in COVID-19 fatalities in comparison to survivors [88]. IgM and IgG against N and S proteins of SARS-CoV-2 were also detected in COVID-19 convalescents [89]. The IgG titer remained high for at least 14 days post-discharge, whereas IgM was detected only in newly recovered patients [89]. Moreover, negative correlation between viral and IgG titers [48] and a significant positive correlation between the content of neutralizing IgG and the number of N protein-specific T cells was observed, suggesting interdependence between humoral and cellular immunity in SARS-CoV-2 infection [48,89].

The kinetics of specific IgG and IgM antibodies were also analyzed in the serum of MERS patients. Robust serological responses were detected in most patients during the second or third week after symptom onset [90,91,92]. Specific IgM antibodies were detectable at the same time or slightly later than IgG [93]. Interestingly, the whole group of survivors, and only half of all fatalities, produced IgG and neutralizing antibodies [91]. Although the MERS-CoV antibody response in the early phase of infection correlated with reduction of the disease severity [90], the presence of antibodies did not allow to the virus removal from the lower respiratory tract [91]. MERS-CoV-specific IgG was also detectable one year post-infection in all severe disease survivors [94]. On the other hand, Alhetheel et al. [95] found a very low rate of MERS-CoV-IgG positive patients and a lack of correlation between nucleic acid and serological analysis [95]. The presence of specific IgA in serum and respiratory tract secretions of MERS patients was also confirmed. Moreover, the correlation between IgA and IgG concentration in serum of MERS-CoV-infected individuals was proven [96,97]. However, as the majority of studies concerning humoral response in MERS used a limited number of patients, using serological analysis is not recommended as a tool to determine disease severity or prognosis.

### 3.2. Differentiation of the Immune Response Depending on HCoV Structural Proteins

Animal models showed that the main antibody responses were induced by the most exposed S protein of SARS-CoV [98,99,100]. Mice immunization with a vector encoding a transmembrane domain of S protein resulted in neutralizing antibody production and action. In consequence, viral replication in the lungs of mice was significantly reduced and immune defense was provided by a humoral but not a T cell-dependent immune mechanism [98]. However, Deming et al. [99] showed that the efficacy of the humoral response to SARS-CoV S protein depended on the homology of the virus strain. Vaccines including a virus replicon expressing SARS-CoV S protein ensured complete short- and long-term protection against homologous strain challenge in young and senescent mice. On the contrary, the implementation of a construct encoding a synthetic S protein gene of the most genetically different human strain resulted in complete short-term protection in vaccinated young mice and limited protection in senescent animals [99].

High tolerance for the vaccine encoding the SARS-CoV S protein and its high immunogenicity has also been documented in humans, with specific antibodies being detected in 80% of subjects [86,101]. Moreover, SARS-CoV-specific CD4^+^ T cell responses were observed in all vaccinated patients and CD8^+^ T cell responses in 20% of individuals [101]. Neutralizing B cell antibody responses to the SARS-CoV S protein were also major in SARS convalescents, suggesting that a spike-based vaccine can be sufficient for a preventive vaccine, as it was previously demonstrated in animal models [40]. As was mentioned above, the strongest response against SARS-CoV S protein was shown by the CD4^+^ T cells. The possible cooperation of CD4^+^ T cell and B cells in neutralizing Ab producing was described previously by Mitchison et al. [102], and the possibility of the enhanced reaction of plasma B cells, stimulated by CD4^+^ T cells, specific to the same protein, has also been suggested [40].

Several studies have documented the presence of antibodies generated against the N protein of SARS-CoV [103,104] and the high affinity of epitopic sites located in the N protein for forming peptide-antibody complexes in the serum of SARS patients, particularly 8 to 14 days after the onset of infection. Interestingly, vaccines containing SARS-CoV N protein failed to protect from homologous and heterologous challenges. In consequence, in the lungs of SARS-CoV-infected mice, the eosinophilic infiltrates were promoted and increased immunopathology was observed [99]. The strongest humoral responses against S and N proteins were also detected in MERS. Although it was proven that MERS-N-specific antibodies occurred later than S-specific antibodies [93], the vaccines containing MERS-specific antibodies are still unknown.

The main features of the humoral response in HCoVs infections are presented in Figure 1.

### 3.3. Antibody-Dependent Enhancement in HCoV Infections

Despite the high range of SARS-CoV-2 infection, the severe course of the disease has mainly affected elderly patients, with children excluded from the risk group [105]. Moreover, despite the high rates of seropositivity of anti-receptor-binding domain (RBD) IgG and IgM (100% and 94%, respectively) and slightly lower rates of anti-N protein IgG and IgM measured after 14 days of symptom onset, the disease was still active and clinical symptoms severe [106]. To explain this phenomenon, antibody-dependent enhancement (ADE) after previous exposure to other HCoVs with a wide range of affinities has been proposed.

In ADE, infection is promoted through a virus binding to non-neutralizing antibodies from previous exposures to similar antigens. The virus–antibody immune complex binds to Fc receptor (FcR) or complement receptors on the host cell surface, facilitating entry of the virus and sometimes enhancing its replication [107,108]. The results of ADE are enhanced inflammatory process, overexpression and release of cytokines (cytokine storm) and multi-organ failure as a consequence of these processes. Immune-mediated CoVs infections have been widely described. The vaccine against feline CoV aggravated future disease via induction of infection-enhancing antibodies [109,110]. Although the full-length SARS-CoV S protein stimulated protective immune response action in rodents, in human B cell lines it induced infection [111]. In vitro studies have demonstrated, that anti-spike immune serum enhanced the infection of immune cells by SARS-CoV Spike-pseudotyped lentiviral particles, as well as replication-competent SARS-CoV, via Fcγ receptor II (FcγRII), but not ACE2 [111,112]. Similarly, Yip et al. [113] documented that human macrophages may be infected by SARS-CoV via FcγRII. However, binding of an immune complex to FcγRII was not sufficient for ADE induction, indicating that activation of the other signaling pathways downstream binding to FcγRII receptors is required [113]. In a SARS-CoV infection of the promonocytic cell line expressing both FcγRII and ACE2, a high concentration of antibodies neutralized the virus, whereas a low content of antibodies induced ADE [114]. Immunization of Rhesus monkeys with a full length SARS-CoV S protein resulted in enhanced disease severity, with a dominant proinflammatory M1-like macrophage profile in the lung tissue, increasing lung injury [110]. Moreover, macrophages produced a significantly greater amount of cytokines in the presence of deceased patients’ serum and SARS-CoV in comparison with the virus alone [115]. Enhanced cytokine production was reduced after FcR blockade. On the contrary, in SARS-CoV the greatest neutralizing antibody titer was observed earlier in deceased patients in comparison with convalescents [116]. However, a recent study showed a new mechanism for ADE by engaging neutralizing antibodies. Monoclonal neutralizing antibody (Mab) binding to the MERS-CoV S protein induced changes in the S protein structure and mediated viral entry to the host cell via IgG FcR [117,118]. Moreover, ADE of MERS-CoV admission depended on the Mab dosage as well as the FcR expression on the cell surface [118].

In humans, besides the immune cells (including monocytes) infiltrating lungs during pneumonia, epithelial cells of the lower respiratory tract also significantly expressed FcγR [119]. In a severe course of SARS and COVID-19 substantial lung opacity was observed, indicating infiltration by monocytes [120,121]. The infection of human monocytes and macrophages by SARS-CoV-2 was also proven [122]. Furthermore, an early humoral response to SARS-CoV-2 and greater antibody titer were positively correlated with a delay in the viral clearance and, in consequence, with the severity of the disease [123]. As mentioned above, great sequence identity and the presence of cross-reactive epitopes of SARS-CoV-2 and other HCoVs were also documented [36,37,124]. Monocyte migration to the lungs and the presence of cross-reactive memory antibodies potentially promote the receptivity of elderly individuals to SARS-CoV-2. The lack of immune memory of closely related HCoVs (and the consequent inability of ADE activation) might be responsible for the absence of clinical symptoms, as well as for the great frequency of undocumented SARS-CoV-2 infection, particularly in children [124]. However, upregulation of ACE2 which is significant component of the renin–angiotensin system (RAS) has also been suggested as a cause of severe courses of HCoVs infections. Animal models demonstrated that angiotensin II receptor type I (AT2R1) antagonists or ACE inhibitors upregulated ACE2 expression [125,126]. Thus, the medicaments widely used in cardiac and hypertensive patients potentially promote the virus binding to the host cells. According to the above, an unequivocal assessment of ADE presence in SARS-CoV-2 infection seems to be crucial in the vaccine development and antibody-based drug therapy. Besides the application of specific anti-SARS-CoV-2, the use of anti-ACE2 with anti-FcγRII monoclonal antibodies to block ADE activation and plasmapheresis for restraining cytokine storm elements in plasma has also been proposed as potentially the most effective method of COVID-19 treatment [127].

## 4. Complement System

The animal model of SARS-CoV infection documented activation of the complement cascade in the lungs and showed that absence of complement significantly reduced the pathological changes in the respiratory tract, even though the viral load is the same. In the lungs of the transgenic mice deficient in C3 (C3^−/−^), which is the central component of the complement system, significantly lower neutrophils and inflammatory monocytes were presented than in infected controls [128]. Moreover, diminished cytokine and chemokine contents in both the lungs and serum of C3^−/−^ mice were detected, suggesting that inhibition of complement signaling might be an efficient therapeutic tool in the treatment of SARS-CoV infection. Similarly, the complement system was inordinately activated in MERS-CoV-infected transgenic mice with human CD26/dipeptidyl peptidase 4 (hDPP4), which is a functional receptor for MERS-CoV. In response to MERS-CoV infection, enhanced contents of C5a and C5b-9 complement activation products in serum and lung tissues of hDPP4-Tg mice, respectively, were observed [129]. Inhibiting C5a production by blocking its receptor (C5aR) reduced lung damage and inflammatory responses [129]. Interestingly, the COVID-19 non-survivors presented lower levels of C3 and C4 proteins at admission in comparison to patients who recovered [88]. Level of C3 protein was suggested as a predictor of mortality of COVID-19 patients.

Recent research showed that mechanisms of responses against HCoVs may also be enhanced by other elements of innate immunity. Activation of human β-defensin 2 (HBD 2) resulted in the conjugation of this protein with the RBD of the MERS-CoV S protein (S RBD) [130,131]. In consequence, expression of chemokines able to recruit leukocytes (comprising monocytes, macrophages, natural killer cells, granulocytes, T cells, and dendritic cells) was promoted. Moreover, enhanced expression of primary immune-inducing molecules (NOD2, TNF-α, IL-1β, and IL-6) and antiviral factors (such as IFN-β, IFN-γ, MxA, PKR, and RNaseL) were also detected, compared to treatment with S RBD alone. Immunization of mice with HBD 2-conjugated S RBD enhanced the immunogenicity of S RBD and induced a stronger S RBD-specific neutralizing antibody response [130]. The S RBD-HBD 2 treatment also increased phosphorylation and activation of receptor-interacting serine/threonine-protein kinase 2 and IFN regulatory factor 3. Moreover, HBD 2 promoted CCR2-mediated Nod2 signaling, inducing the production of type I IFNs and an inflammatory response [131].

Although recognition of the relationship between SARS-CoV-2 infection and complement system activation is extremely important in defining the best path of treatment, the effect of SARS-CoV-2 on complement cascades is still unknown. Li et al. [40] analyzed the association between the cytokine pattern in acute infection and death in SARS and suggested that the quality of immune response, rather than magnitude, may be critical in the progress of the disease. The investigation of innate, humoral, and T cell responses during the critical first 2–3 weeks may indicate whether they require immunosuppressing therapy or not.

## 5. Summary

SARS-CoV-2 has induced the most widespread pandemic in recent decades. The presence of SARS-CoV-2 has resulted in almost 8.5 million COVID-19 patients in 188 countries and territories, including 454,000 fatalities (reported: 18 June 2020 by John Hopkins University). The differing severity of the COVID-19 outbreak has affected different parts of the world for reasons which are still unclear. The epidemiological studies of a previous pandemic, SARS, suggested that human-to-human transmission may enhance the immunogenicity of the virus and its virulence [40]. As SARS-CoV-2 is the first HCoV being transmitted directly among humans, it is highly probable that the wide range of the pandemic is a result of the way of transmission. Although the phylogenetic similarity of SARS-CoV-2 concerned only SARS-CoV and MERS-CoV, but not common HCoVs inducing mild infections of the respiratory system, the potential cross-reactivity of T cells and antibodies between these viruses and its potential impact on total immune responses and clinical outcomes cannot be excluded. Moreover, the presence of mutations in the viral genome is also possible. The next danger is relatively late symptoms occurring in infected people and, in some cases, an asymptomatic course of infection, resulting in a lack of isolation in the early stage of the infection. On the other hand, the genetic similarity of SARS-CoV-2, SARS-CoV, and to a lesser extent to MERS-CoV, the similarities between the structure of the epitopes and receptors, the course of the disease and the effects of the infection, allow undertaking a strategy against COVID-19 based on experience gained during the previous pandemics until the mechanisms of COVID-19 are better understood.

The first studies related to COVID-19 suggested a protective role of both cell-dependent and humoral immune responses in humans. Similar to SARS-CoV and MERS-CoV, the SARS-CoV-2 infection primarily affected T lymphocytes, particularly CD4^+^ and CD8^+^ T cells, resulting in a reduction in their numbers and changes in cytokines secretion, including enhanced IFN-γ production by CD4^+^ T cells. Several studies have also shown the diagnostic utility of serology in SARS, MERS, and COVID-19 investigation. Moreover, the correlation between the severity of the disease and potential immunological markers was documented, which may be a useful prognostic tool of the disease progression, and thereby, in the further course of the pandemic. Based on previous experience, immune-informatic tools were used to define the structure of cytotoxic T lymphocyte and B cell epitopes. However, since SARS-CoV-2 antibody persistence and re-exposure occurrence are still unknown, further studies and a better understanding of the molecular mechanisms of immune responses to SARS-CoV-2 are essential in the new therapeutics development and evaluation of the efficiency of potential vaccines against SARS-CoV-2.

## References

[1] Lai M.M.C., Holmes K.V., Knipe D.M., Howley P.M. (2001). *Coronaviridae*: The viruses and their replication. Fields Virology.

[2] Kahn J.S., McIntosh K. (2005). History and recent advances in coronavirus discovery. Pediatr. Infect. Dis. J..

[3] Masters P.S. (2006). The molecular biology of coronaviruses. Adv. Virus Res..

[4] Neuman B.W., Adair B.D., Yoshioka C., Quispe J.D., Orca G., Kuhn P., Milligan R.A., Yeager M., Buchmeier M.J. (2006). Supramolecular architecture of severe acute respiratory syndrome coronavirus revealed by electron cryomicroscopy. J. Virol..

[5] Barcena M., Oostergetel G.T., Bartelink W., Faas F.G.A., Varkleij A., Rttier P.J.M., Koster A.J., Bosch B.J. (2009). Cryo-electron tomography of mouse hepatitis virus: Insights into the structure of the coronavirion. Proc. Natl. Acad. Sci. USA.

[6] Lai M.M.C., Liao C.L., Lin Y.J., Zhang X. (1994). Coronavirus: How a large RNA viral genome is replicated and transcribed. Infect. Agents Dis..

[7] Malik Y.A. (2020). Properties of Coronavirus and SARS-CoV-2. Malays. J. Pathol..

[8] Fehr A.R., Perlman S. (2015). Coronaviruses: An overview of their replication and pathogenesis. Coronaviruses.

[9] World-Health-Organization Update 49—SARS Case Fatality Ratio, Incubation Period. https://www.who.int/csr/sars/archive/2003_05_07a/en/.

[10] Song Z., Xu Y., Bao L., Zhang L., Yu P., Yajin Q., Zhu H., Zhao W., Han Y., Qin C. (2019). From SARS to MERS, thrusting coronaviruses into the spotlight. Viruses.

[11] Letko M., Marzi A., Munster V. (2020). Functional assessment of cell entry and receptor usage for SARS-CoV-2 and other lineage B betacoronaviruses. Nat. Microbiol..

[12] World-Health-Organization Middle East Respiratory Syndrome Coronavirus (MERS-CoV). https://www.who.int/emergencies/mers-cov/en/.

[13] Zaki A.M., Van Boheemen S., Bestebroer T.M., Osterhaus A.D.M.E., Fouchier R.A.M. (2012). Isolation of a novel coronavirus from a man with pneumonia in Saudi Arabia. N. Engl. J. Med..

[14] Zumla A., Hui D.S., Perlman S. (2014). Middle East respiratory syndrome. Lancet.

[15] Wang D., Hu B., Hu C., Zhu F., Liu X., Zhang J., Wang B., Xiang H., Cheng Z., Xiong Y. (2020). Clinical characteristics of 138 hospitalized patients With 2019 novel coronavirus-infected pneumonia in Wuhan, China. JAMA.

[16] Chan J.F., Kok K.H., Zhu Z., Chu H., To K.K., Yuan S., Yuen K.Y. (2020). Genomic characterization of the 2019 novel human-pathogenic coronavirus isolated from a patient with atypical pneumonia after visiting Wuhan. Emerg. Microbes Infect..

[17] Lu R., Zhao X., Li J., Niu P., Yang B., Wu H., Wang W., Song H., Huang B., Zhu N. (2020). Genomic characterization and epidemiology of 2019 novel coronavirus: Implications for virus origins and receptor binding. Lancet.

[18] Wu F., Zhao S., Yu B., Chen Y.M., Wang W., Song Z., Hu Y., Tao Z., Tian J., Pei Y. (2020). A new coronavirus associated with human respiratory disease in China. Nature.

[19] Gorbalenya A.E. (2020). The species severe acute eespiratory syndrome related coronavirus: Classifying 2019-nCoV and naming it SARS-CoV-2. Nat. Microbiol..

[20] Perlman S., Netland J. (2009). Coronaviruses post-SARS: Update on replication and pathogenesis. Nat. Rev. Microbiol..

[21] Qun L., Xuhua G., Peng W., Wang X., Zhou L., Tong Y., Ren R., Leung K.S.M., Lau E.H.Y., Wong J.Y. (2020). Early Transmission Dynamics in Wuhan, China, of Novel CoronavirusInfected Pneumonia. N. Engl. J. Med..

[22] Burke R.M., Midgley C.M., Dratch A., Fenstersheib M., Haupt T., Holshue M., Ghinai I., Jarashow C.M., Lo J., McPherson T.D. (2020). Active Monitoring of Persons Exposed to Patients with Confirmed COVID-19 – United States, January-February 2020. MMWR Morb. Mortal. Wkly. Rep..

[23] Liu J., Liao X., Qian S., Yuan J., Wang F., Liu Y., Wang Z., Wang F., Liu L., Zhang Z. (2020). Community transmission of severe acute respiratory syndrome coronavirus 2, Shenzhen, China, 2020. Emerg. Infect. Dis..

[24] Lauer S.A., Grantz K.H., Bi Q., Jones F.K., Zheng Q., Meredith H.R., Azman A.S., Reich N.G., Lessler J. (2020). The Incubation Period of Coronavirus Disease 2019 (COVID-19) From Publicly Reported Confirmed Cases: Estimation and Application. Ann. Intern. Med..

[25] Wei W.E., Li Z., Chiew C.J., Yong S.E., Toh M.P., Lee V.J. (2020). Presymptomatic Transmission of SARS-CoV-2—Singapore, 23 January–16 March 2020. MMWR Morb. Mortal. Wkly. Rep..

[26] Zhang X., Hasoksuz M., Spiro D., Halpin R., Wang S., Vlasova A., Janies D., Jones L.R., Ghedin E., Saif L.J. (2007). Quasipecies of bovine enteric and respiratory coronaviruses based on complete genome sequences and genetic changes after tissue culture adaptation. Virology.

[27] Cui J., Li F., Shi Z.L. (2019). Origin and evolution of pathogenic coronaviruses. Nat. Rev. Microbiol..

[28] Ji W., Wang W., Zhao X., Zai J., Li X. (2020). Cross-species transmission of the newly identified coronavirus 2019-nCoV. J. Med. Virol..

[29] Yesilbag Y., Aytogu G. (2020). Coronavirus host divergence and novel coronavirus (Sars-CoV-2) outbreak. CEOTI.

[30] Rabenau H.F., Kampf G., Cinatl J., Doerr H.W. (2005). Efficacy of various disinfectants against SARS coronavirus. J. Hosp. Infect..

[31] Peng H., Yang L., Li J., Lu Z., Wang L., Koup R.A., Bailer R.T., Wu C. (2006). Human Memory T Cell Responses to SARS-CoV E Protein. Microbes Infect..

[32] Dosch S.F., Mahajan S.D., Collins A.R. (2009). SARS Coronavirus Spike Protein-Induced Innate Immune Response Occurs via Activation of the NF-kappaB Pathway in Human Monocyte Macrophages in Vitro. Virus Res..

[33] Aboagye J.O., Yew C.W., Ng O., Manteil V.M., Mirazimi A., Tan Y. (2018). Overexpression of the Nucleocapsid Protein of Middle East Respiratory Syndrome Coronavirus Up-Regulates CXCL10. Biosci. Rep..

[34] Li W., Moore M.J., Vasilieva N., Sui J., Wong S.K., Berne M.A., Samosundaran M., Sullivan J.L., Luzuriaga K., Greenough T.C. (2003). Angiotensin-converting enzyme 2 is a functional receptor for the SARS coronavirus. Nature.

[35] Song W., Gui M., Wang X., Xiang Y. (2018). Cryo-EM structure of the SARS coronavirus spike glycoprotein in complex with its host cell receptor ACE2. PLoS Pathog..

[36] Hasöksüz M., Kiliç S., Saraç F. (2020). Coronaviruses and SARS-COV-2. Turk. J. Med. Sci..

[37] Hoffmann M., Kleine-Weber H., Kruger N., Muller M., Drosten C., Pohlmann S. (2020). The novel coronavirus 2019 (2019-nCoV) uses the SARS-coronavirus receptor ACE2 and the cellular protease TMPRSS2 for entry into target cells. Cell.

[38] Taisheng L., Zhifeng Q., Linqi Z., Yang H., Wei H., Zhengyin L., Xiaojun M., Hongwei F., Wei L., Jing X. (2004). Significant changes of peripheral T lymphocyte subsets in patients with severe acute respiratory syndrome. J. Infect. Dis..

[39] Li T.S., Qiu Z.F., Han Y., Wang Z., Fan H., Lu W., Xie J., Ma X., Wang A. (2003). Rapid loss of both CD4+ and CD8+ T lymphocyte subsets during the acute phase of severe acute respiratory syndrome. Chin. Med. J. (Engl.).

[40] Li C.K., Wu H., Yan H., Ma S., Wang L., Zhang M., Tang X., Temperton N.J., Weiss R.A., Brenchley J.M. (2008). T Cell Responses to Whole SARS Coronavirus in Humans. J. Immunol..

[41] Xie J., Fan H.W., Li T.S., Qiu Z., Han Y. (2006). Dynamic changes of T lymphocyte subsets in the long-term follow-up of severe acute respiratory syndrome patients. Chin. Acad. Med. Sci..

[42] Chu H., Zhou J., Wong B.H., Li C., Chan J.F., Cheng Z., Yang D., Wang D., Lee A.C., Li C. (2016). Middle East Respiratory Syndrome Coronavirus Efficiently Infects Human Primary T Lymphocytes and Activates the Extrinsic and Intrinsic Apoptosis Pathways. J. Infect. Dis..

[43] Shin H., Kim Y., Kim G., Lee J.Y., Jeong I., Joh J., Kim H., Chang E., Sim S.Y., Park J. (2019). Immune Responses to Middle East Respiratory Syndrome Coronavirus During the Acute and Convalescent Phases of Human Infection. Clin. Infect. Dis..

[44] Alosaimi B., Hamed M.E., Naeem A., Alsharef A.A., AlQahtani S.Y., AlDosari K.M., Alamri A.A., Al-Eisa K., Khojah T., Assiri A.M. (2020). MERS-CoV infection is associated with downregulation of genes encoding Th1 and Th2 cytokines/chemokines and elevated inflammatory innate immune response in the lower respiratory tract. Cytokine.

[45] Hong K., Choi J., Hong S., Lee J., Kwon J., Kim S., Park S.Y., Rhee J., Kim B., Choi H.J. (2018). Predictors of Mortality in Middle East Respiratory Syndrome (MERS). Thorax.

[46] Chen G., Wu D., Guo W., Cao Y., Huang D., Wang H., Wang T., Zhang X., Chen H., Yu H. (2020). Clinical and Immunological Features of Severe and Moderate Coronavirus Disease 2019. J. Clin. Investig..

[47] Diao B., Wang C., Tan Y., Chen X., Liu Y., Ning L., Chen L., Li M., Liu Y., Wang G. (2020). Reduction and Functional Exhaustion of T Cells in Patients with Coronavirus Disease 2019 (COVID-19). Front. Immunol..

[48] Weiskopf D., Schmitz K.S., Raadsen M.P., Grifoni A., Okba N.M.A., Endeman H., Van der Akker J.P.C., Molenkamp R., Koopmans M.P.G., Van Gorp E.C.M. (2020). Phenotype and kinetics of SARS-CoV-2-specific T cells in COVID-19 patients with acute respiratory distress syndrome. Sci. Immunol..

[49] Grifoni A., Weiskopf D., Ramirez S.I., Mateus J., Dan J.M., Rydyzynski-Moderbacher C., Rawlings S.A., Sutherland A., Premkumar L., Jadi R.S. (2020). Targets of T Cell Responses to SARS-CoV-2 Coronavirus in Humans with COVID-19 Disease and Unexposed Individuals. Cell.

[50] Baruah V., Bose S. (2020). Immunoinformatics-aided identification of T cell and B cell epitopes in the surface glycoprotein of 2019-nCoV. J. Med. Virol..

[51] Ahmed S.F., Quadeer A.A., McKay M.R. (2020). Preliminary Identification of Potential Vaccine Targets for the COVID-19 Coronavirus (SARS-CoV-2) Based on SARS-CoV Immunological Studies. Viruses.

[52] Channappanavar R., Fett C., Zhao J., Meyerholz D.K., Perlman S. (2014). Virus-specific memory CD8 T cells provide substantial protection from lethal severe acute respiratory syndrome coronavirus infection. J. Virol..

[53] Wen D.G., Pun M.C.K., Chen Z.L., Feng L.Q., Li Z.T., Huang J.C., Ke C.W., Deng X., Ling Y., Wu S.G. (2015). Characteristics of traveler with Middle East respiratory syndrome, China, 2015. Emerg. Infect. Dis..

[54] Veit S., Jany S., Fux R., Sutter G., Volz A. (2018). CD8+ T Cells Responding to the Middle East Respiratory Syndrome Coronavirus Nucleocapsid Protein Delivered by Vaccinia Virus MVA in Mice. Viruses.

[55] Lui P.Y., Won L.Y.R., Fung C.L., Siu K.L., Yeung M.L., Yuen K.S., Chan C.P., Woo P.C.Y., Yuen K.Y., Jin D.Y. (2016). Middle East respiratory syndrome coronavirus M protein suppresses type I interferon expression through the inhibition of TBK1-dependent phosphorylation of IRF3. Emerg. Microbes Infect..

[56] Chang C.H., Liu H.M., Chang M.F., Chang S.C. (2020). Middle East Respiratory Syndrome Coronavirus Nucleocapsid Protein Suppresses Type I and Type III Interferon Induction by Targeting RIG-I Signaling. J. Virol..

[57] Le Bert N., Tan A.T., Kunasegaran K., Tham C.Y.L., Hafezi M., Chia A., Chng M.H.Y., Lin M., Tan N., Linster M. (2020). SARS-CoV-2-specific T cell immunity in cases of COVID-19 and SARS, and uninfected controls. Nature.

[58] Zhang J.M., An J. (2007). Cytokines, inflammation and pain. Int. Anesthesiol. Clin..

[59] Zhou J., Chu H., Li C., Wong B.H., Cheng Z., Poon V.K., Sun T., Lau C.C., Wong K.K., Chan J.Y. (2014). Active Replication of Middle East Respiratory Syndrome Coronavirus and Aberrant Induction of Inflammatory Cytokines and Chemokines in Human Macrophages: Implications for Pathogenesis. J. Infect. Dis..

[60] Kim E.S., Choe P.G., Park W.B., Oh H.S., Kim E.J., Nam E.Y., Na S.H., Kim M., Song K.H., Bang J.H. (2016). Clinical Progression and Cytokine Profiles of Middle East Respiratory Syndrome Coronavirus Infection. J. Korean Med. Sci..

[61] Zhang Y., Li J., Zhan Y., Wu L., Yu X., Zhang W., Ye L., Xu S., Sun R., Wang Y. (2004). Analysis of Serum Cytokines in Patients with Severe Acute Respiratory Syndrome. Infect. Immun..

[62] Zhou P., Yang X.L., Wang X.G., Hu B., Zhang L., Zhang W., Si H., Zhu Y., Li B., Huang C. (2020). A pneumonia outbreak associated with a new coronavirus of probable bat origin. Nature.

[63] Huang C., Wang Y., Li X., Ren L., Zhao J., Hu Y., Zhang L., Fan G., Xu J., Gu X. (2020). Clinical features of patients infected with 2019 novel coronavirus in Wuhan, China. Lancet.

[64] Zlotnik A., Yoshie O. (2000). Chemokines: A New Classification System and Their Role in Immunity. Immunity.

[65] Chen J., Subbarao K. (2007). The Immunobiology of SARS. Annu. Rev. Immunol..

[66] Strayer D., Dickey R., Carter W. (2014). Sensitivity of SARS/MERS CoV to Interferons and Other Drugs Based on Achievable Serum Concentrations in Humans. Infect. Disord. Drug Targets..

[67] Scagnolari C., Trombetti S., Cicetti S., Antonelli S., Selvaggi C., Perrone L., Visca M., Romano S., Antonelli G. (2008). Severe Acute Respiratory Syndrome Coronavirus Elicits a Weak Interferon Response Compared to Traditional Interferon-Inducing Viruses. Intervirology.

[68] Li S., Wang C., Jou Y., Huang S., Hsiao L., Wan L., Lin Y., Kung S., Lin C. (2016). SARS Coronavirus Papain-Like Protease Inhibits the TLR7 Signaling Pathway Through Removing Lys63-Linked Polyubiquitination of TRAF3 and TRAF6. Int. J. Mol. Sci..

[69] Hu W., Yen Y., Singh S., Kao C., Wu-Hsieh B.A. (2012). SARS-CoV Regulates Immune Function-Related Gene Expression in Human Monocytic Cells. Viral Immunol..

[70] Channappanavar R., Fehr A.R., Vijay R., Mack M., Zhao J., Meyerholz D.K., Perlman S. (2016). Dysregulated Type I Interferon and Inflammatory Monocyte-Macrophage Responses Cause Lethal Pneumonia in SARS-CoV-Infected Mice. Cell Host Microbe.

[71] Castilletti C., Bordi L., Lalle E., Rozera G., Poccia F., Agrati C., Abbate I., Capobianchi M.R. (2005). Coordinate Induction of IFN-alpha and -Gamma by SARS-CoV Also in the Absence of Virus Replication. Virology.

[72] Totura A.L., Whitmore A., Agnihothram S., Schafer A., Katze M.G., Heise M.T., Baric R.S. (2015). Toll-Like Receptor 3 Signaling via TRIF Contributes to a Protective Innate Immune Response to Severe Acute Respiratory Syndrome Coronavirus Infection. MBio.

[73] Al-Qahtani A.A., Lyroni K., Aznaourova M., Tseliou M., Al-Anazi M.R., Al-Ahdal M.N., Alkathani S., Sourvinos G., Tsatsanis C. (2017). Middle East Respiratory Syndrome Corona Virus Spike Glycoprotein Suppresses Macrophage Responses via DPP4-mediated Induction of IRAK-M and PPARγ. Oncotarget.

[74] Inn K., Kim Y., Aigerim A., Park U., Hwang E., Choi M., Kim Y., Cho N. (2018). Reduction of Soluble Dipeptidyl Peptidase 4 Levels in Plasma of Patients Infected with Middle East Respiratory Syndrome Coronavirus. Virology.

[75] Law H.K.W., Cheung C.Y., Sia S.F., Chan Y.O., Peiris M., Lau Y.L. (2009). Toll-like Receptors, Chemokine Receptors and Death Receptor Ligands Responses in SARS Coronavirus Infected Human Monocyte Derived Dendritic Cells. BMC Immunol..

[76] Zhao X., Chu H., Wong B.H., Chiu M.C., Wang D., Li C., Liu X., Yang D., Poon V.K., Cai J. (2020). Activation of C-Type Lectin Receptor and (RIG)-I-Like Receptors Contributes to Proinflammatory Response in Middle East Respiratory Syndrome Coronavirus-Infected Macrophages. J. Infect. Dis..

[77] Gorse G.J., Donovan M.M., Patel G.B. (2020). Antibodies to coronaviruses are higher in older compared with younger adults and binding antibodies are more sensitive than neutralizing antibodies in identifying coronavirus-associated illnesses. J. Med. Virol..

[78] Li G., Chen X., Xu A. (2003). Profile of specific antibodies to the SARS-associated coronavirus. N. Engl. J. Med..

[79] Chen W.J., Xu Z.Y., Mu J.S., Yang L., Gan H., Mu F., Fan B., He B., Huang S., You B. (2004). Antibody response and viraemia during the course of severe acute respiratory syndrome (SARS)-associated coronavirus infection. J. Med. Microbiol..

[80] He Z., Dong Q., Zhuang H., Song S., Peng G., Luo G., Dwyer D.E. (2004). Kinetics of Severe Acute Respiratory Syndrome (SARS) Coronavirus-Specific Antibodies in 271 Laboratory-Confirmed Cases of SARS. Clin. Diagn. Lab. Immunol..

[81] Hsueh P.R., Huang L.M., Chen P.J., Kao C.L., Yang P.C. (2004). Chronological evolution of IgM, IgA, IgG and neutralisation antibodies after infection with SARS-associated coronavirus. Clin. Microbiol. Infect..

[82] Ksiazek T.G., Erdman D., Goldsmith C.S., Zaki S.R., Peret T., Emery S., Tong S., Urbani C., Corner J.A., Lim W. (2003). A novel coronavirus associated with severe acute respiratory syndrome. N. Engl. J. Med..

[83] Peiris J.S., Chu C.M., Cheng V.C.C., Chan K.S., Hung I.F.N., Poon L.L.M., Law K.I., Tang B.S.F., Hon T.Y.W., Chan C.S. (2003). Clinical progression and viral load in a community outbreak of coronavirus-associated SARS pneumonia: A prospective study. Lancet.

[84] Mo H., Xu J., Ren X., Zeng G., Tan Y., Chen R., Chan-Yeung M., Zhong N. (2005). Evaluation by Indirect Immunofluorescent Assay and Enzyme Linked Immunosorbent Assay of the Dynamic Changes of Serum Antibody Responses Against Severe Acute Respiratory Syndrome Coronavirus. Chin. Med. J. (Engl.).

[85] Tang F., Quan Y., Xin Z.-T., Wrammert J., Ma M.-J., Lv H., Wang T.-B., Yang H., Richardus J.H., Liu W. (2011). Lack of peripheral memory B cell responses in recovered patients with severe acute respiratory syndrome: A six-year follow-up study. J. Immunol..

[86] Cao Z., Liu L., Du L., Zhang C., Jiang S., Li T., He Y. (2010). Potent and persistent antibody responses against the receptor-binding domain of SARS-CoV spike protein in recovered patients. Virol. J..

[87] Hou H., Wang T., Zhang B., Luo Y., Mao L., Wang F., Wu S., Sun Z. (2020). Detection of IgM and IgG antibodies in patients with coronavirus disease 2019. Clin. Transl. Immunol..

[88] Zhao Y., Nie H.X., Hu K., Wu X.J., Zhang Y.T., Wang M.M., Wang T., Zheng Z.S., Li X.C., Zeng S.L. (2020). Abnormal immunity of non-survivors with COVID-19: Predictors of mortality. Infect. Dis. Poverty.

[89] Ni L., Ye F., Cheng M.L., Feng Y., Deng Y.Q., Zhao H., Wei P., Ge J., Gou M., Li X. (2020). Detection of SARS-CoV-2-Specific Humoral and Cellular Immunity in COVID-19 Convalescent Individuals. Immunity.

[90] Park W.B., Perera R.A.P.M., Choe P.G., Lau E.H.Y., Choi S.J., Chun J.Y., Oh H.S., Song K.H., Bang J.H., Kim E.S. (2015). Kinetics of Serologic Responses to MERS Coronavirus Infection in Humans, Korea. Emerg. Infect. Dis..

[91] Corman V.M., Albarrak A.M., Omrani A.S., Albarrak M.M., Farah M.E., Almasri M., Muth D., Sieberg A., Meyer B., Assiri A.M. (2016). Viral Shedding and Antibody Response in 37 Patients With Middle East Respiratory Syndrome Coronavirus Infection. Clin. Infect. Dis..

[92] Al-Kahlout R.A., Nasrallah G.H., Farag E.A., Wang L., Lattwein E., Muller M.A., El-Zowalaty M.E., Al-Romaihi H.E., Graham B.S., Al-Thani A.A. (2019). Comparative Serological Study for the Prevalence of Anti-MERS Coronavirus Antibodies in High- And Low-Risk Groups in Qatar. J. Immunol. Res..

[93] Wang W., Wang H., Deng Y., Song T., Lan J., Wu G., Ke C., Tan W. (2016). Characterization of anti-MERS-CoV antibodies against various recombinant structural antigens of MERS-CoV in an imported case in China. Emerg. Microbes Infect..

[94] Choe P.G., Perera R.A.P.M., Park W.B., Song K., Bang J.H., Kim E.S., Kim H.B., Ko L.W.R., Park S.W., Kim N. (2017). MERS-CoV Antibody Responses 1 Year After Symptom Onset, Korea, 2015. Emerg. Infect. Dis..

[95] Alhetheel A., Altalhi H., Albarrag A., Shakoor Z., Mohamed D., El-Hazmi M., Somily A., Barry M., Bakhrebah M., Nassar M. (2017). Assessing the Detection of Middle East Respiratory Syndrome Coronavirus IgG in Suspected and Proven Cases of Middle East Respiratory Syndrome Coronavirus Infection. Viral Immunol..

[96] Muth D., Corman V.M., Meyer B., Assiri A., Al-Masri M., Farah M., Steinhagen K., Lattwein E., Al-Tawfig J.A., Albarrak A. (2015). Infectious Middle East Respiratory Syndrome Coronavirus Excretion and Serotype Variability Based on Live Virus Isolates from Patients in Saudi Arabia. J. Clin. Microbiol..

[97] Ko J.H., Muller M.A., Seok H., Park G.E., Lee J.Y., Cho S.Y., Ha Y.E., Baek J.Y., Kim S.H., Kang J.M. (2017). Suggested new breakpoints of anti-MERS-CoV antibody ELISA titers: Performance analysis of serologic tests. Eur. J. Clin. Microbiol. Infect. Dis..

[98] Yang Z., Kong W., Huang Y., Roberts A., Murphy B.R., Subbarao K., Nabel G.J. (2004). A DNA vaccine induces SARS coronavirus neutralization and protective immunity in mice. Nature.

[99] Deming D., Sheahan T., Heise M., Yount B., Davis N., Sims A., Suthar M., Harkema J., Whitmore A., Pickles R. (2006). Vaccine efficacy in senescent mice challenged with recombinant SARS-CoV bearing epidemic and zoonotic spike variants. PLoS Med..

[100] Hu H., Lu X., Tao L., Bai B., Zhang Z., Chen Y., Zheng F., Chen J., Chen Z., Wanh H. (2007). Induction of Specific Immune Responses by Severe Acute Respiratory Syndrome Coronavirus Spike DNA Vaccine with or Without interleukin-2 Immunization Using Different Vaccination Routes in Mice. Clin. Vaccine Immunol..

[101] Martin J.E., Louder M.K., Holman L.A., Gordon I.J., Enama M.E., Larkin B.D., Andrews C.A., Vogel L., Koup R.A., Roederer M. (2008). A SARS DNA Vaccine Induces Neutralizing Antibody and Cellular Immune Responses in Healthy Adults in a Phase I Clinical Trial. Vaccine.

[102] Mitchison N.A. (2004). T-cell-B-cell cooperation. Nat. Rev. Immunol..

[103] Wang J., Wen J., Li J., Yin J., Zhu Q., Wang H., Yang Y., Qin E., You B., Li W. (2003). Assessment of immunoreactive synthetic peptides from the structural proteins of severe acute respiratory syndrome coronavirus. Clin. Chem..

[104] Liu X., Shi Y., Li P., Li L., Yi Y., Ma Q., Cao C. (2004). Profile of antibodies to the nucleocapsid protein of the severe acute respiratory syndrome (SARS)-associated coronavirus in probable SARS patients. Clin. Vaccine Immunol..

[105] Peron J.P.S., Nakaya H. (2020). Susceptibility of the Elderly to SARS-CoV-2 Infection: ACE-2 Overexpression, Shedding, and Antibody-dependent Enhancement (ADE). Clinics.

[106] To K.K., Tsang O.T., Leung W.S., Tam A.R., Wu T.C., Lung D.C., Yip C.C., Cai J.P., Chan J.M., Chik T.S. (2020). Temporal profiles of viral load in posterior oropharyngeal saliva samples and serum antibody responses during infection by SARS-CoV-2: An observational cohort study. Lancet Infect. Dis..

[107] Tirado S.M., Yoon K.J. (2003). Antibody-dependent enhancement of virus infection and disease. Viral Immunol..

[108] Takada A., Kawaoka Y. (2003). Antibody-dependent enhancement of viral infection: Molecular mechanisms and in vivo implications. Rev. Med. Virol..

[109] Vennema H., De Groot R.J., Harbour D.A., Dalderup M., Gruffydd-Jones T., Horzinek M.C., Spaan W.J. (1990). Early death after feline infectious peritonitis virus challenge due to recombinant vaccinia virus immunization. J. Virol..

[110] Hohdatsu T., Yamada M., Tominaga R., Makino K., Kida K., Koyama H. (1998). Antibody-dependent enhancement of feline infectious peritonitis virus infection in feline alveolar macrophages and human monocyte cell line U937 by serum of cats experimentally or naturally infected with feline coronavirus. J. Vet. Med. Sci..

[111] Kam Y.W., Kien F., Roberts A., Cheung Y.C., Lamirande E.W., Vogel L., Chu S.L., Tse J., Guarner J., Zaki S.R. (2007). Antibodies against trimeric S glycoprotein protect hamsters against SARS-CoV challenge despite their capacity to mediate FcgammaRII-dependent entry into B cells in vitro. Vaccine.

[112] Jaume M., Yip M.S., Cheung C.Y., Leung N.H.L., Li P.H., Kien F., Dutry I., Callendret B., Escriou N., Altmeyer R. (2011). Anti-severe acute respiratory syndrome coronavirus spike antibodies trigger infection of human immune cells via a pH- and cysteine protease-independent FcγR pathway. J. Virol..

[113] Yip M.S., Leung N.H.L., Cheung C.Y., Li P.H., Yeung H.H., Daeron M., Peiris J.S.M., Bruzzone R., Jaume M. (2014). Antibody-dependent infection of human macrophages by severe acute respiratory syndrome coronavirus. J. Virol..

[114] Wang Q., Zhang L., Kuwahara K., Li L., Liu Z., Li T., Zhu H., Liu J., Xu Y., Xie J. (2016). Immunodominant SARS Coronavirus Epitopes in Humans Elicited both Enhancing and Neutralizing Effects on Infection in Non-human Primates. ACS Infect. Dis..

[115] Liu L., Wei Q., Lin Q., Fang J., Wang H., Kwok H., Tang H., Nishiura K., Peng J., Tan Z. (2019). Anti-spike IgG causes severe acute lung injury by skewing macrophage responses during acute SARS-CoV infection. JCI Insight.

[116] Zhang L., Zhang F., Yu W., He T., Yu J., Yi C.E., Ba L., Li W., Farzan M., Chen Z. (2006). Antibody responses against SARS coronavirus are correlated with disease outcome of infected individuals. J. Med. Virol..

[117] Du L., Zhao G., Yang Y., Qiu H., Wang L., Kou Z., Tao X., Yu H., Sun S., Tseng C.T. (2014). A conformation-dependent neutralizing monoclonal antibody specifically targeting receptor-binding domain in Middle East respiratory syndrome coronavirus spike protein. J. Virol..

[118] Wan Y., Shang J., Sun S., Tai W., Chen J., Geng Q., He L., Chen Y., Wu J., Shi Z. (2020). Molecular Mechanism for Antibody-Dependent Enhancement of Coronavirus Entry. J. Virol..

[119] The Protein Cell Atlas Webpage. https://www.proteinatlas.org/ENSG00000143226-FCGR2A/tissue.

[120] Guan W.J., Ni Z.Y., Hu Y., Liang W.H., Ou C.Q., He J.X., Liu L., Shan H., Lei C.L., Hui D.S. (2020). Clinical Characteristics of Coronavirus Disease 2019 in China. N. Engl. J. Med..

[121] Ooi G.C., Daqing M. (2003). Sars: Radiological features. Respirology.

[122] Bost P., Giladi A., Liu Y., Bendjelal Y., Xu G., David E., Bletcher-Gonen R., Cohen M., Medaglia C., Li H. (2020). Host-viral infection maps reveal signatures of severe COVID-19 patients. Cell.

[123] Tan W., Lu Y., Zhang J., Wang J., Dan Y., Tan Z., He X., Qian C., Sun Q., Hu Q. (2020). Viral kinetics and antibody responses in patients with COVID-19. MedRxiv.

[124] Fierz W., Waltz B. (2020). Antibody Dependent Enhancement Due to Original Antigenic Sin and the Development of SARS. Front. Immunol..

[125] Ferrario C.M., Jessup J., Chappell M.C., Averill D.B., Brosnihan K.B., Tallant E.A., Diz D.I., Gallagher P.E. (2005). Effect of angiotensin-converting enzyme inhibition and angiotensin II receptor blockers on cardiac angiotensin-converting enzyme 2. Circulation.

[126] Huang M.L., Li X., Meng Y., Xiao B., Ma Q., Ying S.S., Wu P.S., Zhang Z.S. (2010). Upregulation of angiotensin-converting enzyme (ACE) 2 in hepatic fibrosis by ACE inhibitors. Clin. Exp. Pharmacol. Physiol..

[127] Sedokani A., Feizollahzadeh S. (2020). Plasmapheresis, Anti-ACE2 and Anti-FcγRII Monoclonal Antibodies: A Possible Treatment for Severe Cases of COVID-19. Drug Des. Dev. Ther..

[128] Gralinski L.E., Sheahan T.P., Morrison T.E., Menachery V.D., Jensen K., Leist S.R., Whitmore A., Heise M.T., Baric R.S. (2018). Complement Activation Contributes to Severe Acute Respiratory Syndrome Coronavirus Pathogenesis. MBio.

[129] Jiang Y., Zhao G., Song N., Li P., Chen Y., Guo Y., Li J., Du L., Jiang S., Guo R. (2018). Blockade of the C5a-C5aR Axis Alleviates Lung Damage in hDPP4-transgenic Mice Infected With MERS-CoV. Emerg. Microbes Infect..

[130] Kim J., Yang Y.L., Jang S., Jang Y. (2018). Human β-Defensin 2 Plays a Regulatory Role in Innate Antiviral Immunity and Is Capable of Potentiating the Induction of Antigen-Specific Immunity. Virol. J..

[131] Kim J., Yang Y.L., Jang Y. (2019). Human β-Defensin 2 Is Involved in CCR2-mediated Nod2 Signal Transduction, Leading to Activation of the Innate Immune Response in Macrophages. Immunobiology.

